# Making Sense of Missense: Assessing and Incorporating the Functional Impact of Constitutional Genetic Testing

**DOI:** 10.3390/children12111449

**Published:** 2025-10-24

**Authors:** Meaghann Weaver

**Affiliations:** 1 Bioethics Program, St. Jude Children’s Research Hospital, Memphis, TN 38105, USA; meaghann.weaver2@stjude.org; 2 Division of Palliative Care, St. Jude Children’s Research Hospital, Memphis, TN 38105, USA

**Keywords:** pediatric oncology, cancer predisposition, Li-Fraumeni syndrome

## Abstract

**Highlights:**

**What are the main findings?**

- Assessing families for cancer predisposition syndromes is an increasingly common practice in pediatric oncology.
- Testing for cancer-predisposition syndromes in pediatric oncology enables cancer surveillance, helps tailor and inform cancer-directed treatments, and may assist in family risk assessment.

**What is the implication of the main finding?**

- Receiving a diagnosis of a cancer predisposition syndrome can be a complex experience with practical, existential, and psychosocial implications for patients and their families.
- Consideration should be given to each family’s experience in receiving a cancer predisposition diagnosis, for both their immediate processing of the diagnosis and subsequent need for longitudinal support.

**Abstract:**

**Background/Objectives**: With the emergence of accessible and affordable next-generation sequencing platforms, pediatric oncologists are now accountable to diligently ascertain genetic causes of cancer, with an amenable opportunity to test for cancer predisposition syndromes. **Methods**: This study incorporates triangulated interviews of family members diagnosed with Li–Fraumeni syndrome through clinical *TP*53 testing. The interview content was coded using NVivo 10.0 software to determine psychosocial themes relevant to genetic testing, diagnosis, and surveillance. **Results**: Interview content revealed opportunities to apply social themes analogous to *TP53* biologic language. **Conclusions**: This report models the systematic inclusion of patient, parent, and health care provider perspectives when testing individuals for familial cancer predisposition syndromes.

## 1. Introduction

A pre-teen male was diagnosed with acute myeloid leukemia after having been treated for localized osteosarcoma 5 years earlier. The family history contained a complex array of primary and secondary cancers in extended family members on the maternal line. The patient met Chompret criteria for Li–Fraumeni syndrome (LFS) and therefore underwent genetic testing for *TP*53 mutations. The patient harbored a *TP*53 mutation. His acute myeloid leukemia was recognized as high risk based on myelodysplastic features and 7q- and monosomy 5 karyotype, thereby expediting a bone marrow donor search. The patient’s mother and sister also had a *TP*53 mutation, thus excluding them from donating.

The tumor-suppressor gene *TP53* represents a common denominator among human cancers [1]; therefore, the mechanism of *TP53* action may serve as an analogy for an approach to genetic testing. With the emergence of accessible and affordable next-generation sequencing platforms, pediatric oncologists are now accountable to diligently ascertain the genetic causes of cancer with an amenable opportunity to test family members for cancer predisposition syndromes. The p53 protein guards the genome by regulating uncontrolled, hazardous cell division, thereby preventing harmful tumor formation. The clinical approach to testing for germline mutations may best be viewed as a guardian role—identifying individuals at risk of cancer and initiating tumor surveillance represents a protective extension of patient care. Just as p53 is in the cell nucleus, where it binds directly to DNA, pediatric oncologists attentively care for the child with cancer (“the nucleus”) while maintaining close therapeutic alliance with the patient’s family (“the cell”). *TP53* encodes a transcription factor that is activated by cellular stress to minimize the propagation of harm [2]; therefore, the clinical team must approach constitutional genetic testing in a way that programmatically coordinates the antiproliferation of psychosocial stress, activation of support, and minimization of harm. Although recent evidence endorses tumor surveillance in pediatrics, this is not yet a universal practice [3,4]. Each individual and their family bring their own story and experiences, which motivate a methodological investment in eliciting narratives to guide future interventions for genetic testing, diagnosis, and overall care.

Receiving a cancer predisposition diagnosis is a life-altering experience that can compel a continuum of either negative factors of blame, guilt, and family instability or the positive factors of resilience, growth, and empowerment through knowledge. Concern about the psychological burden of genetic testing on families has been explored extensively in adult-onset cancer predisposition syndromes [5,6], but it is mostly speculative in pediatrics [7]. LFS models the destructive, divisive effects of accumulating damage; thus, families carrying germline *TP53* mutations may experience a high risk of intrapersonal distress [8]. Although transient psychological anxiety may occur, long-standing, clinically significant distress need not be experienced when protective health delivery checkpoints are in place [9,10]. For genetic syndromes like LFS, with well-known medical benefits of strategic monitoring [3,4,11,12], the value of surveillance in the hope of detecting tumors at an early stage has been accompanied by a greater sense of control, security, and empowerment for families [13]. Caution should be contextually applied when discussing the perceived clinical benefits of surveillance, as those benefits may be more robust for some diagnoses (comprehensive LFS surveillance protocols) than others in which evidence is limited, lacking, or population-specific (e.g., founder variants in a particular geographical setting).

The relevance of genetic testing in this case was for personalized risk assessment and tailored treatment strategies. Psychosocial considerations included family counseling and cascade testing. The main research rationale was to determine how communication about cancer predisposition testing and diagnoses could improve prognostic understanding and enhance psychosocial adjustment to the news. Investigating patient- and family-specific motivations for and responses to genetic testing has the potential to translate into protective clinical checkpoints and improved psychosocial endpoints [14].

## 2. Materials and Methods

This qualitative study was developed through a protocol titled LiSTENING (Learning in Story: Elicited Narrative Informs Navigating Genetics). The Institutional Review Board at St. Jude Children’s Research Hospital approved this study under expedited categories, and informed consent was obtained from all study participants.

The LiSTENING protocol elicited patient and family narratives to inform interventions for genetic testing, diagnosis, and overall care. This qualitative approach mapped family members’ interpretation of their experience receiving a diagnosis of a germline *TP*53 mutation. This family-based qualitative case study enabled the study team to learn about potential psychosocial risks and benefits of genetic testing from a triangulated perspective of the patient, family, and care provider [15].

The family (*n* = 4 interviewed members) was recruited through study referral from the primary oncology clinic. The first approach included individual interpretations, as heard during family members’ recorded personal semi-structured interviews. The second approach included a collective perspective obtained through patient/parent/family semi-structured interviews. The bounded lens of this research was then interpreted through professional reflection with the multidisciplinary care providers. This approach enabled the acquisition of a detailed medical history, the social context, the chronology of events (both proximally and distally), and the interpretation of events from diverse vantage points [16]. Although the quantity of data gathered during interviews restricted its institutional implementation, targeted use of qualitative investigation enables teams to clarify themes and explore interpretations not readily accessible through quantitative methods [17,18].

This study followed strict qualitative investigation standards in study design (Appendix A.1) and implementation (Appendix A.2) [19]. Interview questions were first fielded by two qualitative research teams, one genetic counselor, one cancer psychologist, and were piloted on adult patients with cancer harboring a *BRCA1* mutation (Appendix A.3). As a stakeholder participatory study, interviewees were given the opportunity to review and refine the interview transcripts prior to final data coding. Using an inductive approach to data analysis, interview words were used as the elements of data, and interview content was coded utilizing NVivo qualitative analysis software [20].

The field of genetics relies on meticulous coding; therefore, the interviews were attentively coded for social constructs. Coding reliability was ensured through inter-rater reliability (>94% for each domain), with differences in initial perspectives discussed until a consensus was reached. Social constructs were then interpreted into genetic language, thereby enabling social scientists and medical scientists to analyze and apply important themes via a shared language.

## 3. Results

Interview content revealed an opportunity to apply social themes analogous to *TP53* biologic language (Table 1). Biological parents, one sibling, and one patient were offered the opportunity to participate in the qualitative interviews, as part of this in-depth original case study. The patient and parents emphasized the importance of the care team’s role to “check in” and elicit decisional preferences prior to each step in the diagnostic pathway: decision to undergo testing, receipt of results, and planning for surveillance. Before deciding about genetic testing, the family was asked to consider the ethical and relational aspects of disclosing test results to extended relatives.

Before testing, the care team had determined the family’s preferences for who would be present for conversations about the initial diagnosis. Because genetic findings would affect the person’s candidacy for bone marrow donor, each family member was pre-emptively encouraged to consider their coping with the test results, not as an isolated medical finding but as part of a larger care context. Thus, the team conducted conversations about testing, resulting, and monitoring prior to the testing to foster readiness. Mutated health system checkpoints could result in the proliferation of harm (e.g., testing without adequate partnership or preparation), or the other extreme––systematic blockage despite the potential benefits of genetic knowledge.

Comments to discourage genetic testing (“that’s so rare, it’s not worth testing,” or “most people don’t want to know”) were perceived by the family as imposing “selective pressure,” and yet they considered genetic testing a right and a responsibility. Regulatory checkpoints are best informed by clinical relations, as insight into family history and social context not only helps protect patients and families but also fosters safe progression toward the diagnosis of cancer predisposition syndromes.

The interviews revealed individual and collective stages of adjustment to the news of cancer predisposition, an adjustment equally important for framing the past and anticipating the future. The initial stress and shock of the diagnosis threatened personal growth arrest, requiring family members to individually and then collectively progress beyond the initial “devastation” into adjustment and acceptance. The family found strength in a unified interpersonal front, sharing poignant examples of aligning with one another (senescence) to face tensions and challenges. The theme of repair was depicted as repairing knowledge by correcting one another’s misinformation, repairing attitudes of guilt (carrier) and blame (noncarrier), and repairing perceptions that fear of a future cancer should steal today’s joys. Finding out about cancer predisposition was described as providing improved psychological adjustment to past loss, providing a new frame of reference for past events (frame shift).

The family history of multiple, concurrent cancer diagnoses and premature deaths was interpreted differently after germline discovery. The family’s imagination had previously leaned toward interpretations of loss as a scary and uncontrolled plight, whereas science now provided a cause that spoke reason to their past experience. Secret worries about what one family member termed a “mutant” or “freaky” extended family medical history could now be contained within a context of scientific explanation.

The most compelling insight gained during the interviews was the dominant theme of gain of function through genetic insight. Interviewees spoke of confidence and reduced uncertainty resulting from pre-symptomatic screening exams. Empowerment for advocacy, scientific knowledge, and validation of past suspicions were perceived as gains for older family members. Planning, self-knowledge, and early maturity were depicted as gains of functions for younger family members. The interviewees stated their goal of limiting the effect of cancer by discovering any future cancer early. For extended family members, monitoring was perceived as a gain of function, as targeted surveillance enabled them to believe cancer was now less likely “to sneak up, to surprise” them at an advanced stage.

**Figure 1:**
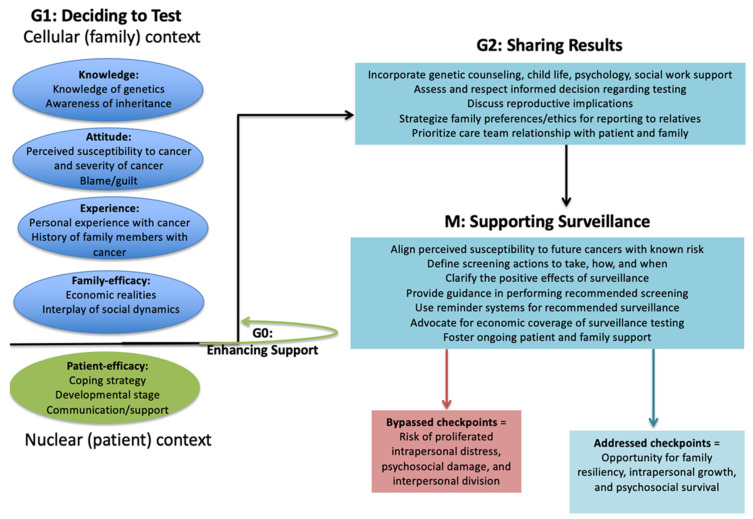
Conceptual model: role of clinical checkpoints in genetic testing. The first Gap (G1) checkpoint assesses the patient’s and family’s knowledge, attitude, belief, and efficacy prior to their key decision of whether to undergo genetic testing. This checkpoint may result in promptly proceeding to testing or delay of testing (G0). For some individuals, further educational/social support may be needed before they proceed. The G2 checkpoint helps maintain family stability by assessing support interventions to ensure the family is ready to learn their results. Also, G2 may trigger an increase in family support to avoid interpersonal divisions and personal damage and to maintain family stability. The Metaphase (M) checkpoint involves continued alignment of support when sharing news with extended relatives and engaging in tumor surveillance (times of possible tension) to maintain personal and family integrity.

By iteratively engaging with content from the interviews, the multidisciplinary study team formulated a conceptual model that supports patients and families through constitutional genetic testing (Figure 1). The figure applies the phases of a cell cycle to model a parallel psychosocial process for patients and families undergoing genetic testing. Genetic language translation helped connect the family’s lived narrative with the treatment team’s biomedical insights and the scientific team’s existing language. Genetic testing represents the science of medicine. Psychosocial engagement represents the art of medicine. Medicine merges science and art at the bedside. Translating genetic language into psychosocial principles fostered shared language as an opening for additional communication.

## 4. Discussion

This paper adds to the pediatric oncology literature by transforming the idea of cancer predisposition communication with families from a one-size-fits-all approach to a more precise, preventive, and family-centered discipline. The methods and findings from this paper demonstrate how cancer predisposition communication can inform treatment and support families.

Cancer predisposition testing warrants attention to ethical safeguards, beyond high-quality informed consent processes, that clearly communicate the purpose, benefits, risks, limitations, and alternatives to genetic testing; the voluntary nature of testing, free from coercion; and the right to withdraw or not receive test results. Additional ethics considerations include equitable access to genetic counseling before and after testing to review the implications, possible outcomes, and options, and to conceptualize the next steps after interpreting the test results. Protocols for providing emotional support to participants who receive distressing results should be part of a comprehensive care model.

Cancer predisposition results carry familial implications, which include not only the weight of self-determination but also the responsibilities associated with relational autonomy. Patients benefit from guidance on sharing relevant results with at-risk relatives, while also respecting their relatives’ individual autonomy. Ethical dilemmas arise in balancing patient confidentiality with the potential benefit of informing at-risk relatives. Therefore, ethics consultation services should be available and accessible in the setting of cancer predisposition testing. Cultural humility, fair access, and equity principles remain paramount to ensuring that the availability of cancer predisposition testing transcends economic barriers, while remaining tailored to cultural and linguistic needs. Privacy and confidentiality remain key health system ethics considerations with attentiveness to protecting data and disclosure. From a community perspective, legal protections that safeguard against genetic discrimination, in terms of insurance coverage and employment, have an increased importance in this era of genetic testing. Although cancer predisposition may be tested in one individual, the results of that testing have ethical implications across families; thus, psychosocial support needs are magnified.

This study explored the complex phenomena within one family, with specific attention to real-life contexts and contextual analysis. The methods included applying a biology metaphor to frame the psychosocial data. One limitation of metaphors is that they are used to compare unlike things, which can be ambiguous. Not all complex concepts, definitions, or arguments can be captured metaphorically, but metaphors can help foster understanding by illustrating such topics in a less complex way.

Subjective interpretation of the qualitative narrative risks researcher bias. Emotional recall, social desirability, and family dynamics risk participant bias, especially in the context of discussing sensitive genetic diagnoses. A limitation of this study was the single-site, single-family unit size, which prevents the generalizability of our findings. The in-depth, personalized engagement with the family demonstrated an exemplary approach for each family’s unique needs.

As pediatric oncology teams prepare to systematically approach genetic testing, there is an increasing, urgent need to gather the insight of those with lived experience. Understanding the nature of modifying effects, as well as the biological context in which they occur, has fostered new approaches aimed at controlling the clinical-biological impact of *TP53* mutation [21]. Similarly, understanding genetic testing motivations and social context has the potential to promote innovative approaches to supporting clinical–social adjustments to cancer predisposition diagnoses.

The perspectives of those who have undergone cancer predisposition testing have the potential to teach care teams how to maximize psychosocial growth and minimize harm in the process. When DNA interfaces with potential harm, p53 plays an essential role in determining whether the DNA will be repaired or the organism will suffer. De-mystifying genetic testing, encouraging provider transparency, and unifying silos of interest are key to enabling clinical medicine to catch up with the speed at which potentially beneficial cancer predisposition data are being generated via next-generation sequencing. The first step to this end is the thoughtful inclusion of stakeholders’ perspectives, which may inform this era of genetic testing and speak sense to missense.

## Figures and Tables

**Table 1:** Psychosocial constructs relevant to genetic principles.

Genetic Principle	Psychosocial Construct	Exemplifying Quote
Missense Point mutation with impact on function	Poignant moment of receiving positive constitutional genetic test results, subsequently trying to “make sense” of life in context of cancer predisposition.	“Well, I don’t remember too much about that actual day of hearing the genetic results. Maybe I choose not to.” “It was upsetting … it was devastating to the whole family when you hear something like this [genetic diagnosis]”
Checkpoint activation Protective monitoring of cell progression through sequential events	Process of care team confirming patient and family preparation prior to sequential testing events: deciding to undergo diagnostic test (G1), receiving test result (G2), initiating surveillance testing (M).	G1—“She [genetics counselor] was, in a way, kind of persuading us not to do genetic testing because she said, “Well, most people don’t want to know.” I think that is her job, you know, making sure families are prepared and ready for the news, of course. I know they [children] are very young, but our family needs to know. We have a right to know. It is important for our health to know.” G2—“If something else was to go wrong with someone in our family, that now we can look closer because of the genetic importance … it’s important … we needed to get information from the test result. They prepared us to get results.” M—“Although, the extra testing is both the easiest part and the hardest part. Like, I mean, they need to come here and get some more scans and things that we weren’t going to be doing before. I will need to do that too, for my health. And, from my point of view, a hard part is paying the doctor or hospital for testing and imaging and stuff that you normally wouldn’t be spending money or insurance bills on since now from the genetics you are going to have these test done. But, the easiest part would be that maybe you could find a different cancer sooner and start treatment earlier.”
Frameshift insertion Altered reading of codons due to new insertion	Impact on a person’s interpretation of family medical history due to new frame of reference (input of genetics knowledge altering translation of events).	“When I was in my early twenties, my father had a brain tumor and he saw a specialist, of course. After they had removed some of the brain tumor, they did some testing, and they said that they thought that it might be a genetic possibility for the fact of how large the brain tumor was and the location. But he never actually got tested, even though, well, he also had leukemia later. So I was always, in the back of my mind, was always wondering, ‘what is this?’” “I asked about osteosarcoma and whether it could be genetic way back years ago, when he had his first cancer, and we came for his first treatment. I asked about genetics way back then … and they told me no. They told me it was just something that happened. So, you know, maybe at that time if genetics was brought up then we would have thought about it more and done genetic testing back then. Maybe, maybe we would have known about our family genes sooner. Maybe knowing about the genetics back then would have helped us find and diagnose this second cancer sooner.”
Modifier Effectual influence on the expression of another gene	Recognition that the way one family member processes and responds to a genetic diagnosis may influence psychosocial experience of another family member.	“I agree with what she [mom] says … what she says influences how I feel and think about the genetics.”
Alternative Splicing Single gene coding for multiple proteins with differences in sequence and function	Acknowledgement of the unique personality of each family member, warranting personalized approach to timing and content of communication about genetic testing (single message, multiple interpretations).	“He’s quite mature on a lot of the medical things being the fact that everything he’s gone through at such an early age. So, he needed different words in the genetic talks.” “For her, it’s different, because she’s still very young and she wants children, but it’s also very confusing. And she, as a sister, has to see this, so she’s kind of a little conflicted. Whether to even think of having her own children and not getting the whole scope or thinking the whole scope of everything. We have to talk to her in that unique way of thinking further down the line and her future.” “They have different needs. They need to hear things differently. And, they both think differently.”
Alternate promoters Privileged region of transcription initiation	Recognition that certain care team members or family members may be better positioned than others to initiate essential tasks such as deciding to undergo testing or result sharing.	“For them [certain relatives], of course, there was no problem explaining the genetic news to them. And then, of course, with them it’s a very real possibility that they have it too and so I felt an obligation to tell. I told.” “So I thought and thought and whether to tell them [certain distant relatives] about the cancer gene. It was very hard to just make a decision if I should contact them and tell them this or not. After much thinking, I decided since I don’t have a very close relationship with them, that I didn’t have a role to tell them. And I had left it with my grandfather and decided that he was closer to them than I was and that if he wanted to make the decision to tell them, he could. And I left it that way. I think that was best because he knows them and their personal needs to know or not to know.”
Gain of function Neomorphic mutation, resulting in gene product with increased function	Gain of insight or perceived benefit from receipt of genetic diagnosis; a dominant theme.	“You get lumps or bumps or moles or little things and you just think, “oh, it’s no big deal.” And it really could be something. So, you know, try to find out [genetic results] as soon as possible, it’s a better option, I think, when trying to get treatment you can get it earlier and protect yourself.” “Personally, I would want to know if there’s something wrong and what I can do to prevent it or to find out sooner just in order to be able to live a healthy, happy life with my children. So, to me … it’s just like going to the dentist and finding out you have a cavity. You need to get it filled now, or you wait and then you need a root canal. But, the best thing is to find out about genetics early so you can help your family.”
Loss of function Amorphic mutation, resulting in gene product with less function	Concern about loss of personal or family function due to genetic test result; a recessive theme.	“Well, if you think about it, a cancer could happen anytime, anytime. A cancer could happen just out of nowhere. Cancer could happen again to you, or the cancer could happen to someone you love. Cancer could come again to your family because of the genes.” “These are hard life decisions, and I am having to think about these choice earlier than most people. Well, everyone has to make that very important life choice. That choice is if you want to still have kids or just stop even thinking about it and don’t have kids at all. I will have to think about genes when I think about that choice.”
Growth arrest Cessation of progression in cell cycle	Feelings of guilt, harm, stress, or stagnation due to damaging interactions or shock of familial cancer predisposition.	“But then, knowing that he’s gone through all this, as a mother’s point, you kind of feel like you might not want to have even ever had children because you don’t want them to suffer through this.” “When my dad was sick, the thought “maybe that is also in my DNA” had not occurred to me yet. Of course, by the time we actually knew there was something really, really wrong in our family health history, I was already pregnant with my daughter. But, I mean, if I knew then what I know now, I probably wouldn’t have had children. I would have thought not to have children, I mean, to save him from all of this very hard journey. I watch him. It’s hard.”
Senescence Cessation in division	Finding strength in family unity and comfort in togetherness; resistance to separation.	“I have a sister, and I try to talk to hear about it since we both have it … it’s something we share.” “We should hear all news together. I want for us to be together. We can face this better when we are together.”
Transactivation activities Increased rate of gene expression	Increasing expression of strength in knowledge.	“Personally, for me, I think I wanted to know [genetic predisposition] for my future, for my health, and for my well-being.” “Knowing is important for our daughter’s health, too. I know, little things, to take her to the doctor and be more aggressive with the doctor. As in, you know, for my son, it took two or three times for his first diagnosis. And his second diagnosis, I took him I don’t know how many times to our family doctor, ‘something’s wrong.’ But now, I would be adamantly like, ‘no, I know there is something wrong and these are the possibilities.’ If that makes sense. Knowing about the genes make me feel like I have an obligation to seek care and to get medical answers.”
Repair Cell identification of potential damage with corrective mechanisms	Fixing misinformation, mending misinterpretations, and providing new information through science	“Of course, my daughter wasn’t sure exactly what all the genetics language meant and she made the comment, ‘does that mean I have cancer?’ right away when they told her about the positive genetic test. Because, you know … she didn’t really understand the whole logic of the genetics, of course. I knew right away, and I said, ‘No, that’s not what the genetics test meant. The cancer gene doesn’t mean you have cancer.’ And, of course, they [the doctors] are great, they assured her, ‘No, no. That’s not what that means. It just means that your DNA’s a little bit different.’ They explained in a way that she could understand that this was meant to help for them to monitor her closer, but, it doesn’t mean that she’s going to for sure have cancer. It just means that she might have an increased possibility of having cancer … more possibility than a person normally would be. So, that helped. My daughter had fears, and they used their words to clarify and calm her fears.” “It’s kind of a vague thing, it’s like they [medical community] don’t know much about it. I kind of really feel like there’s actually more people out there that have it. I mean, how many people wake up and say, “Gee, I want to get DNA tested to see if I have a cancer gene or a migraine gene?” You know? So I think that there’s a lot more people out there. It would help me to know there are more people with this mutation. Maybe more people getting tested could make it feel more normal for the people who do get tested.”
Silent mutation	Resistance to allowing the genetic result to impose guilt, to change personal functions, to negatively impact view cancer, to change family structure, or to harm lived experiences.	“It, really, for me, it’s not a … not a horrible burden. It’s the way you’re made, it was nothing that you did wrong or anything like that. I mean, you feel bad because you have passed it to somebody else, especially since they, my children, have gone through so very much and I haven’t. But, like I said, it’s nothing anybody did wrong.” “I don’t think or live like I’m a ticking time bomb.” “I don’t think of the cancer any different knowing that genes caused it. He’s still sick. We still have to have treatment. It’s cancer. There’s no difference if cancer is genetic or not genetic because the treatment is not different at this time.” “I’m not disappointed that we did the genetic testing. In fact, I’m happy that we did it. To me, it’s not any different than going to the doctor and having them do some lab work and saying, ‘you’re diabetic now.’ If that makes sense, it should be treated like any other medical test. Some medical tests are important … you know, a pregnancy test, for example. You’re pregnant, you’re not pregnant, but it’s always just a test. The test impacts the future. What you do with the test is what is important. But, the test itself is no different than any other medical test.” “You know that you might live just a perfect life and never have another cancer. Your family might never have a cancer. You can’t be scared just because of genes.” “Life goes on. It’s not the end of the world to find out [about LFS].” “You could have totally different types of cancer. But, you continue with your daily life. It’s not like your life has stopped because of a genetic diagnosis, I mean, ours hasn’t.”

## Data Availability

The original contributions presented in this study are included in the article. Further inquiries can be directed to the corresponding author.

## Abbreviations

The following abbreviations are used in this manuscript:

G0–G2	Gap0, Gap1, and Gap2 are the cellular growth phases
LFS	Li–Fraumani syndrome
M	Metaphase
*TP53*	Tumor protein 53 gene

## Appendix A

### Appendix A.1. Quality Tactics Specific to Research Design

**Quality Measure**	**Research Tactic Used**	**Applied**	**Timing of Tactic**
Construct validity	- Used multiple sources of evidence - Established chain of evidence - Engaged in iterative process of investigation	- Individual informants, family unit informants, multidisciplinary care team informants - Detailed review of written records and oral narrative - Continued return to primary sources - Provided drafts of report to key informants	- Data collection - Composition
Internal validity	- Matched patterns - Built explanations - Addressed rival explanations	- Used logic models and conceptual frameworks	- Data analysis
External validity	- Used theory	- Incorporated elements of Health Belief Models and Theory of Reasoned Action	- Research design
Reliability	- Used study protocol - Developed study database	- LiSTENING protocol, IRB approved - Developed study database using NVivo 10.0 software)	- Design - Data collection - Composition
	Table revised with permission from Yin, R.K. *Research: Design and Methods, 5th edition* (SAGE Publications, Inc., 2013), page 45 [15].		

### Appendix A.2. Consolidated Criteria for Reporting Qualitative Research (COREQ)

**Domain 1. Research team and reflexivity**	
**Personal characteristics**	
1. Interviewer/facilitator	MW conducted the interviews
2. Credentials	The study team consisted of doctoral-level investigators from the field of pediatric oncology, genetics, and psychology
3. Occupation	Members of the study team included oncologists, a geneticist, a laboratory investigator, and a pediatric psychologist
4. Gender	The research team consisted of a balance of gender representation
5. Experience and training of interviewer	The interviewer has completed cognitive interview graduate-level training from sociology/anthropology investigators, has completed qualitative software coursework with tutelage from an Atlas.ti software designer, completed graduate-level qualitative methodology coursework prior to study design and interview implementation, and receives ongoing mentoring from qualitative investigators
**Relationship with participants**	
6. Relationship established	The study was mentioned to the family by their psychologist and oncologist two weeks prior to interviews with an in-person introduction to interviewer occurring one day prior to interviews
7. Participant knowledge of interviewer	The participants were made aware that the interviewer is interested in decision-making constructs and that the study team was interested in learning about the family’s experiences to gain insight about impacts of genetic testing
8. Interviewer characteristics	The interviewer utilizes positive affirmation during interviews (nodding and sharing verbal agreement) with follow-up questions (while avoiding leading) and tolerates extended silence during interviews to foster open, engaged conversations
**Domain 2. Study design**	
**Theoretical framework**	
9. Methodological orientation and theory	Qualitative study methodology underpinned the study; inductive reasoning (grounded theory) was applied for data analysis
**Participant selection**	
10. Sampling	The participants were selected due to complexity of medical history and recognized importance of learning from their health experiences (purposive sampling approach)
11. Method of approach	Participants were approached face-to-face by interviewer
12. Sample size	*n* = 2 individuals, *n* = 1 family unit
13. Non-participation	Two invited participants elected to participate after an engaged process of informed consent
**Setting**	
14. Setting of data collection	Data were collected in clinic conference room setting, which is a comfortable room with sofa chairs (non-medical environment)
15. Presence of non-participants	No one else was present during interviews besides the participants and researchers; the participants were given the option of including non-participants or their psychologist in the room for support
16. Description of sample	Demographics and data are deliberately omitted to protect the privacy/confidentiality of participants
**Data collection**	
17. Interview guide	The questions were reviewed by a genetic-psychologist expert from a different institution, two genetic counselors, and two qualitative research teams piloted on an adult cancer patient diagnosed with a *BRCA*1 mutation
18. Repeat interviews	Repeat interviews were carried out, as per the triangulated format described in methodology
19. Recording	Interviews were voice recorded
20. Field notes	Field notes were not made during the interviews, but, the interview engaged in memoing immediately after the interviews to reflect upon interview setting/content
21. Duration	The interviews each lasted just under one hour
22. Data saturation	Saturation was not an a priori goal; any and all raised themes were included as relevant
23. Transcripts returned	Transcripts were returned to the participants for comments and for opportunity for correction
Domain 3. Analysis and findings	
24. Number of data coders	Two data coders coded the data
25. Description of coding tree	Coding tree provided as table within manuscript
26. Derivation of themes	Themes were identified from the data as social constructs and two data coders then converted the social constructs into genetic terminology
27. Software	NVivo was used to manage the data
28. Participant checking	Participants provided feedback on findings
**Reporting**	
29. Quotations presented	Participant quotations were presented to illustrate themes. To protect participant privacy, quotation was not identified by participant name.
30. Data and findings consistent	The data presented were consistent with evidence base gathered through literature search completed prior to interview initiation
31. Clarity of major themes	Major themes were clearly presented in the form of text summary
32. Clarity of minor themes	Minor themes were described by list format in the manuscript figure
	Adapted with permission from Tong, A., Sainsbury, P. & Craig, J. Consolidated criteria for reporting qualitative research (COREQ): a 32-item checklist for interviews and focus groups. *Int. J. Qual. Health Care* 19, 349–357 (2007) [19].

### Appendix A.3. Interview Script for LiSTENING Study Protocol

**Timepoint 1. Questions for Individual Family Member Interviews**

*We would like to learn from you as a _____ [insert relevant word: patient, sibling, or parent]. If you feel like the way I am asking a question does not fit your experience or does not best describe your idea for the question, please let me know better words to use for your experience—or, let me know if you want to skip that part. Although we discussed this as part of the consent process, we do wish to remind you that because of the unique medical circumstances, it cannot be guaranteed that your shared responses will be de-identified (meaning: someone reading a report of the responses could guess it was you answering the questions if they previously knew your medical history). We welcome you to share your story. We have interview questions available, if you feel the questions would help to walk through a discussion of your experience.*

*If we bring up an idea that doesn’t exist for you, you can correct us or we can skip that part of the interview. As we discussed as part of the consent process, we do wish to again remind you that because of the unique medical circumstances, it cannot be guaranteed that your shared responses will be de-identified (meaning: someone reading a report of the responses could guess it was your family answering the questions if they previously knew the medical history of your family).*

When did you first learn about the genetic diagnosis?

What did it feel like?

What questions did you have about the genetic diagnosis?

Who did you talk to?

Anybody you thought you couldn’t talk to?

Do you talk to your doctor about it?

Are there things you wanted to know that still haven’t been answered?

[*This question is for the parents only*]—What were your thoughts about talking to your children about the test results? Did you want to? Did you feel ready to? How did you decide what to say?

When do you think about it the most?

Looking back, if you had control of the past, when do you think the best timing to receive this information would be—as in, would you have wanted to know forever or not until you were a certain age or have wanted to never know?

What difference did this make in your family’s life?

What difference did this make in your life?

How has receiving the genetic news impacted you, personally?

Based on your life experiences, do you have recommendations for how I could approach genetic testing or information about genetic diagnoses for _____ *[insert: patients, siblings, dads, or moms according to interviewee identity]* in my future interactions?

Is there anything I have not asked you which you would like to share?

*Interviewer then thanks study participant for the generous gift of time and responses.*

**Timepoint 2. Questions for Family Unit Interview Session**

*We are interested to learn more your experience with genetic testing as a family so that we (as care teams) may learn from your insight. You have some experiences that other people maybe don’t have. But, with more families undergoing genetic testing, more people will likely receive news of cancers being associated with a genetic diagnosis. We would therefore wish to learn from you about what it has been like and maybe learn what things have gone well and what things can go better. If we bring up an idea that doesn’t exist for you, you can correct us or we can skip that part of the interview. As we discussed as part of the consent process, we do wish to remind you that because of the unique medical circumstances, it cannot be guaranteed that your shared responses will be de-identified (meaning: someone reading a report of the responses could guess it was your family answering the questions if they previously knew the medical history of your family). We welcome you to share your story. We have interview questions available, if you feel the questions would help to walk through a discussion of your experience.*

What is the beginning, as in, when did you first hear that cancer may be associated with a genetic diagnosis? (*Interviewer pauses and then repeats the word “beginning” after stating the question to focus the family to a starting chronology context.*)

What did this mean to you?

What stands out from your communication amongst one another in deciding to undergo genetic testing?

What are your thoughts about cancers being hereditary or having a familial component?

What were you feeling or experiencing while you decided to undergo genetic testing?

What were your feelings while you waited for the results (from the time of deciding to undergo testing to the time of hearing results)?

What was it like for you to receive results?

Was everyone in the family together at the same time to hear the diagnosis—or, who was there?

[*If everyone was not present for initial diagnosis*]—What was your experience like sharing this information with each other?

Were the genetic results different than you expected? If so, in what ways?

When you heard the genetic results, what were your initial thoughts or considerations?

How did these thoughts or considerations change or evolve over time?

What questions did you have for your health care team?

Are there any questions that you still have—questions that are maybe unanswered?

What has it been like for you since you received the genetic results?

What has changed in your feelings since you learned this information?

What has changed in your lives?

How do you talk about it? Who do you talk about it with?

What is different now as compared to when you first heard?

Through the whole journey, what were the hardest parts?

I don’t want to impose a certain positive perception if this does not exist, but, I am wondering if are there certain parts that you found to be positive or memorable in a way that was good for your family?

For some genetic diagnosis, there is the opportunity for surveillance or screening tests (such as more frequent exams). What are your thoughts about the experience of earlier screening?

Tell me about whether you decided to share results with extended relatives and how you came to that decision?

[*If the family states that they did decide to share*]—How did you go about this? What have been reactions?

As a health care community, we try hard to think about experiences and to reflect on things that went well and also to acknowledge things that may have been better. What would you like physicians or others to learn?

Are there ways the medical community could have been more helpful?

What was it like for you when people were maybe particularly sensitive to your family’s needs, or, even helpful?

What do you think would be the best way to counsel cancer patients and their families in preparation for genetic testing?

What should I know, as a pediatric oncologist in training, about the impact of genetic testing on families?

Is there anything I have not asked you which you would like to share or which you feel is important for me to know?

*Interviewer then thanks family for their generous gift of time and for their very thoughtful responses in teaching her about their experiences.*
